# Supplementary material for: Two new species of fossil Leggadina (Rodentia: Muridae) from Northwestern Queensland
Source: PeerJ. 2015 Jul 9;3:e1088. doi: 10.7717/peerj.1088 (PMC4511818; doi:10.7717/peerj.1088)
Supplement: Supplemental Information 1 [file peerj-03-1088-s001.docx]

Table S1: **A complete list of fossil specimens used in this study.**

| **ID** | **Specimen Type** | **Location** |
| --- | --- | --- |
| QM F57240 | M^1^, right | Rackham’s Roost Site, Riversleigh WHA, QLD |
| QM F57241 | M^1^, left, partial maxillary | Rackham’s Roost Site, Riversleigh WHA, QLD |
| QM F57242 | M^1^, right | Rackham’s Roost Site, Riversleigh WHA, QLD |
| QM F57243 | M^1^, right | Rackham’s Roost Site, Riversleigh WHA, QLD |
| QM F57244 | M^1^, right, partial maxillary | Rackham’s Roost Site, Riversleigh WHA, QLD |
| QM F57245 | M^1^, left | Rackham’s Roost Site, Riversleigh WHA, QLD |
| QM F57246 | Upper molar row, right, partial maxillary | Rackham’s Roost Site, Riversleigh WHA, QLD |
| QM F57247 | M^2^, left, partial maxillary | Rackham’s Roost Site, Riversleigh WHA, QLD |
| QM F57248 | M^1^ and M^2^, right, partial maxillary | Rackham’s Roost Site, Riversleigh WHA, QLD |
| QM F57249 | M^1^ and M^2^, right, partial maxillary | Rackham’s Roost Site, Riversleigh WHA, QLD |
| QM F57250 | M^1^ and M^2^, right | Rackham’s Roost Site, Riversleigh WHA, QLD |
| QM F57251 | M^1^ and M^2^, right | Rackham’s Roost Site, Riversleigh WHA, QLD |
| QM F57252 | M^1^ and M^2^, left | Rackham’s Roost Site, Riversleigh WHA, QLD |
| QM F57253 | Upper molar row, right, partial maxillary | Rackham’s Roost Site, Riversleigh WHA, QLD |
| QM F57254 | M^1^, left | Rackham’s Roost Site, Riversleigh WHA, QLD |
| QM F57255 | M^1^, left, partial maxillary | Rackham’s Roost Site, Riversleigh WHA, QLD |
| QM F57256 | M^1^ and M^2^, right, partial maxillary | Rackham’s Roost Site, Riversleigh WHA, QLD |
| QM F57257 | M^1^ and M^2^, left, partial maxillary | Rackham’s Roost Site, Riversleigh WHA, QLD |
| QM F57258 | M^1^ and M^2^, left, zygomatic plate | Rackham’s Roost Site, Riversleigh WHA, QLD |
| QM F57259 | Upper molar row, right, partial maxillary | Rackham’s Roost Site, Riversleigh WHA, QLD |
| QM F57260 | Upper molar row, right | Rackham’s Roost Site, Riversleigh WHA, QLD |
| QM F57261 | M^1^, right | Rackham’s Roost Site, Riversleigh WHA, QLD |
| QM F57262 | Upper molar row, right | Rackham’s Roost Site, Riversleigh WHA, QLD |
| QM F57263 | M^1^ and M^2^, right | Rackham’s Roost Site, Riversleigh WHA, QLD |
| QM F57264 | M^1^, left, partial maxillary | Rackham’s Roost Site, Riversleigh WHA, QLD |
| QM F57265 | M^1^, right, partial maxillary | Rackham’s Roost Site, Riversleigh WHA, QLD |
| QM F57283 | Upper molar row, left | Rackham’s Roost Site, Riversleigh WHA, QLD |
| QM F39958 | Upper molar row, left | Rackham’s Roost Site, Riversleigh WHA, QLD |
| QM F57266 | M^1^, right | Site 5C, Floraville Station, QLD |
| QM F57267 | M^1^, left | Site 5C, Floraville Station, QLD |
| QM F57268 | M^1^, left | Site 5C, Floraville Station, QLD |
| QM F57269 | M^1^, left | Site 5C, Floraville Station, QLD |
| QM F57270 | M^1^, right | Site 5C, Floraville Station, QLD |
| QM F57271 | M^1^, left | Site 5C, Floraville Station, QLD |
| QM F57272 | M^1^, right | Site 5C, Floraville Station, QLD |
| QM F57273 | M^2^, left | Site 5C, Floraville Station, QLD |
| QM F57274 | M^1^, left | Site 5C, Floraville Station, QLD |
| QM F57275 | M^1^, left, partial maxillary | Site 5C, Floraville Station, QLD |
| QM F57276 | M^1^, left, zygomatic plate | Site 5C, Floraville Station, QLD |
| QM F57277 | M^1^, left | Site 5C, Floraville Station, QLD |
| QM F57278 | M^1^, right | Site 5C, Floraville Station, QLD |
| QM F57279 | M^1^, right | Site 5C, Floraville Station, QLD |
| QM F57280 | M^1^, right | Site 5C, Floraville Station, QLD |
| QM F57281 | M^1^, right | Site 5C, Floraville Station, QLD |
| QM F57282 | M^1^, left | Site 5C, Floraville Station, QLD |
