# Supplementary figures and images for: Two new species of fossil Leggadina (Rodentia: Muridae) from Northwestern Queensland
Source: PeerJ. 2015 Jul 9;3:e1088. doi: 10.7717/peerj.1088 (PMC4511818; doi:10.7717/peerj.1088)

Table S2.1: **Univariate statistics for fossil murid specimens.** Measurements to 2 decimal places
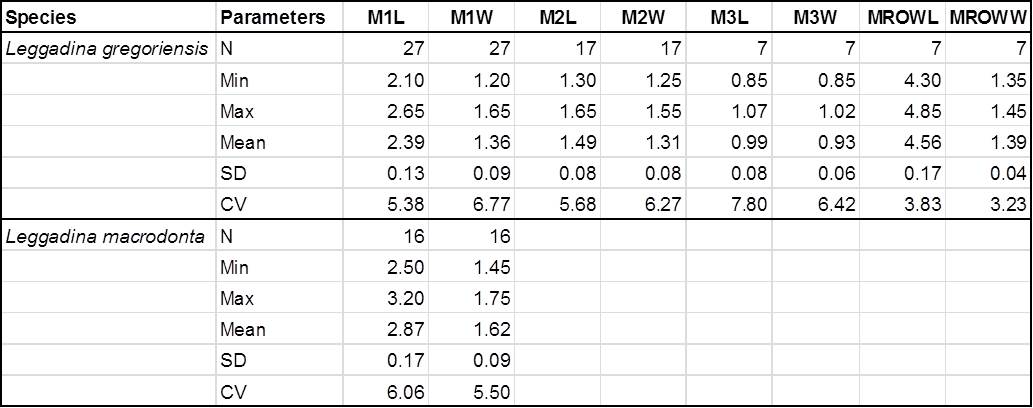

Supplement: Supplemental Information 2 — Measurements to 2 decimal places. [file peerj-03-1088-s002.docx]
